# Influence of Gamma Irradiation and Water Aging on the Space Charge Characteristics of Epoxy Micro-Nano Composites

**DOI:** 10.3390/polym13060964

**Published:** 2021-03-22

**Authors:** Myneni Sukesh Babu, Ramanujam Sarathi, Takahiro Imai, Toshikatsu Tanaka

**Affiliations:** 1 Department of Electrical Engineering, Indian Institute of Technology Madras, Chennai 600036, India; ee18d003@smail.iitm.ac.in; 2 Infrastructure Systems Research and Development Center, Toshiba Infrastructure Systems & Solutions Corporation, Toshiba-cho, Fuchu-shi, Tokyo 183-8511, Japan; takahiro2.imai@toshiba.co.jp; 3 Electrical and Electronics Department, IPS Research Centre, Waseda University, Kitakyushu 808-0135, Japan; t-tanaka@waseda.jp

**Keywords:** epoxy, micro-nano composites, silica, ion-trapping nano particle, gamma irradiation, water aging, space charge

## Abstract

Epoxy micro-nano composites are well-known to exhibit enhanced electrical, mechanical as well as thermal properties compared to base epoxy resin. Yet, a clear understanding need to be achieved on the long-term aging performance of the epoxy micro-nano composites. The present review article is a comprehensive study on the impact of gamma irradiation and water aging on the space charge characteristics of epoxy micro-nano composites that are applicable as insulant in high-voltage power apparatus. Ion-trapping nanoparticles, which possess good oxidation resistance and high ion trapping ability, are being chosen as nanofillers along with silica micro fillers in epoxy micro-nano composite material for improving the reliability of electrical insulation structures. The epoxy micro-nano composite specimens were subjected to gamma irradiation (4 kGy and 8 kGy) and water aging (under room temperature and at 90 °C), to analyze the effect of aging on space charge accumulation and charge decay characteristics. The mean magnitude of accumulated space charge density of epoxy micro-nano composites tends to increase with an increase in gamma irradiation dose as well as an increment in water diffusion coefficient. The mean lifetime of the space charge decay during depoling has significantly reduced after gamma irradiation and is converse with water aged specimen. Voltage polarity reversal studies have indicated that a part of homo-charge injected from electrodes remained as hetero-charge just after polarity reversal and could result in the distortion of electric field thereby increasing the electric field enhancement factor.

## 1. Introduction

In recent years, polymeric nanocomposite insulating materials such as epoxy nanocomposites are gaining attention to be used as an insulant in power apparatus like rotating machines, cast resin dry-type transformers, and as spacers in Gas Insulating Switchgear (GIS) [1,2,3]. Because of the high surface area of the fillers, it is possible to achieve improved properties such as higher volume resistivity, higher breakdown strength, lower space charge accumulation, and lower dielectric loss by reinforcing small quantities of micro or nano particles into the epoxy matrix [4,5,6,7]. The electrical, mechanical, and thermal properties of polymeric nanocomposites depend on the type of polymer matrix and filler, their physical properties such as size, shape, and weight percentage of the fillers [8,9,10,11,12,13]. Proper dispersion of the micro or nano particles in the polymer matrix also plays a crucial role in the improvement of desired properties of the composite material [14]. Hence, it is essential to determine the optimal weight percentage of filler in a polymer matrix.

A considerable amount of work has been carried in the recent studies to understand the behavior of epoxy resin by adding micro or nano fillers such as clays (montmorillonite), inorganic oxides such as silica, alumina, titania, magnesia, zinc oxide, etc., and metal particles such as aluminum, nickel, and silver [15,16,17,18,19,20,21,22,23,24]. Imai et al. have stated that the silica micro particles exhibit low coefficient of thermal expansion [25]. In addition, Goyanes et al. have stated that the addition of the silica micro fillers tends to improve storage modulus along with inhibiting thermal expansion coefficient of the composites [26]. Tsekmes et al. have indicated that the epoxy micro-nano composites can have the advantages of higher breakdown strength and improved thermal conductivity [27]. The use of 60–70 wt.% of micro-sized silica particles as filler in epoxy composite insulators is a general trend in industry [27]. Mishra et al. have studied about the corona discharges initiated due to water droplets on ion-trapping nano particle-filled epoxy nanocomposites. An ion-trapping nano particle is a hydrotalcite compound modified with zirconium phosphate. Inorganic ion exchangers like zirconium phosphate tend to exhibit good oxidation resistance along with high ion trapping ability. Hence, it can be applicable in the electrical insulation structures for improving their reliability [28]. Furthermore, it can enhance the thermal capability of the insulating material, because of the presence of zirconium, a refractory material [29]. Additionally, the study on the influence of reinforcing inorganic ion exchangers into epoxy-silica micro composites, in order to use them as insulant, is discussed very little [29,30], and a proper database needs to be established.

The exposure of polymeric insulating materials to various stresses during their usage may deteriorate its properties and may lead to its early degradation [31,32,33,34,35]. In radiation environments such as nuclear power plants and space equipment, the application of epoxy insulating materials is increasing rapidly [28,36]. Hence, it is necessary to analyze the performance of the epoxy nanocomposites with respect to different levels of gamma-ray irradiation. The ionizing radiations can alter the molecular structure of polymeric insulating materials through different physical and chemical mechanisms like cross-linking, chain scissions, and oxidation [37]. These structural changes induced due to the exposure of gamma radiation tends to alter the space charge and charge trap characteristics of insulating material [38]. One of the major concerns in epoxy nanocomposite insulating material is the tendency of water uptake in humid environments. Water diffused into the epoxy composite specimens is present in the free volume of the micro cavities in the matrix and can also present as bound water, which forms hydrogen-bonding with polar segments of the material [39]. This phenomenon is severe in case of composites compared to base epoxy matrix, as the matrix-filler interface acts as a potential site for the water molecules to interact [40]. It can also lead to irreversible material changes such as plasticization and swelling [41]. Epoxy specimens are subjected to water aging at high temperatures in order to simulate the accelerated ageing and to understand the long-term consequences of water intake phenomenon [42].

Polymeric insulating materials under high electric fields tend to accumulate charge in the volume of the specimen. This accumulated space charge can alter the internal electric field, resulting in the early degradation of insulating material [43,44,45,46]. Noah et al. have indicated that the DC electric fields above 3 kV/mm can trigger space charge accumulation in the epoxy resin [47]. The voltage polarity reversal phenomenon in general happens in HVDC transmission systems for achieving bi-directional power flow. The presence of space charge during the voltage polarity reversal can become a vital threat to the insulation cables in HVDC system [48,49]. Chen et al. have indicated that the dosage of gamma radiation as well as ambience of radiation can significantly affect the space charge accumulation in the insulating material [50]. Montanari et al. have stated that the presence of humidity can enhance space charge accumulation, which can possibly affect the charge trapping sites [51]. The water shell model proposed by Zou et al., which is based on Lewis’ [52] and Tanaka′s [53] models, explains the effect of water absorption in epoxy nanocomposites when they are exposed to humidity [40]. If the water concentration around the nanoparticles is high, percolative paths are formed through overlapping water shells that can affect the dielectric properties of epoxy nanocomposites [47]. So, it is essential to understand the influence of gamma radiation and water aging on the space charge accumulation under different electric fields and during polarity reversal. Various methods have been employed to measure the space charge in the insulating material including thermal step method (TSM), Laser Induced Pressure Pulse (LIPP), Pulse Electro Acoustic (PEA), Pressure Wave Propagation (PWP), etc., and each method has its own advantages and disadvantages. Of these, the Pulse Electro Acoustic (PEA) method is one of the most reliable and promising technique for the measurement of space charge [54]. Hence, in the present study, PEA method of space charge measurement has been employed.

Therefore, the prime focus of the present review paper is to assess the long-term aging performance and to establish a proper database on the influence of various aging conditions on the space charge accumulation and charge decay characteristics of epoxy micro-nano composites, under positive DC electric field and during voltage polarity reversal phenomenon.

## 2. Experimental Setup and Aging Procedure

### 2.1. Specimen Details

**Figure 1:**
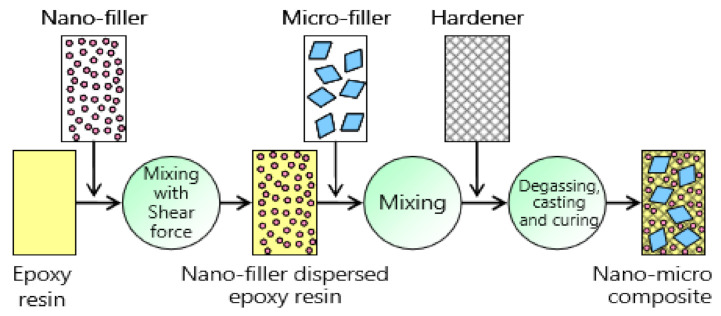
Scheme for the preparation of epoxy micro-nanocomposites [30].

The epoxy micro-nano composite specimen, which consists of base epoxy resin reinforced with crystalline SiO_2_ micro fillers (66 wt. %) ion-trapping nano particles as nano fillers (0.7 wt. %). The SiO_2_ micro fillers and the ion-trapping nano fillers have an average diameter of 14 µm and 200–500 nm, respectively. Surface modification of fillers by silane coupling agent has been employed. Epoxy micro-nanocomposites specimens were prepared by following the standard shear mixing, degassing, casting, and curing procedures as shown in Figure 1 [29].

### 2.2. Aging Procedure

The test epoxy specimens were subjected to gamma-ray irradiation through a ^60^Co radiation source in air ambience with a dosage rate of 660 Gy/h up to total dosage of 4 kGy and 8 kGy. Hence, three test specimens unaged, 4 kGy and 8 kGy gamma irradiated specimens were selected for analysis. Epoxy specimens have been immersed in deionized water at 90 °C and room temperature for 240 h, respectively. The percentage weight gain of epoxy specimens can be calculated as shown in (1).

(1) $$P\left(t\right)=\frac{W\left(t\right)-W_{O}}{W_{O}}\times 100\%$$

where $W_{O}$ is the mass of unaged specimen, *W(t)* is the mass of test specimen at an ageing time *t*, and *P(t)* is the percentage weight gain of the epoxy sample at an aging time *t*.

**Figure 2:**
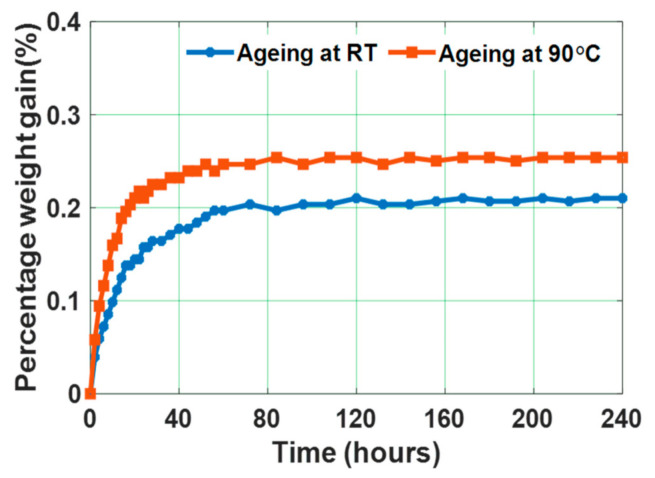
Percentage weight gain of epoxy samples subjected to water ageing at room temperature (RT) and 90 °C [30].

Figure 2 depicts the percentage weight gain of water aged test samples, aged at room temperature and at 90 °C separately. The weight gain becomes almost constant by 240 h. The initial water diffusion rate and the final weight gain are higher for 90 °C water aged specimens compared to room temperature water aged specimens. The water diffusion coefficient can be determined as shown in (2) [30].

(2) $$D=\frac{\pi L_{0.5}^{2}}{64t_{0.5}^{\;}}$$

where *L* is the thickness of sample, *D* is the diffusion coefficient, and *t*_0.5_ is time required for the specimen to reach half of the steady state value of percentage weight gain.

The water diffusion coefficients of the test samples aged in deionized water at room temperature and 90 °C are 4.97 × 10^−12^ m^2^ s^−1^ and 7.74 × 10^−12^ m^2^ s^−1^, respectively. Diffusion of water is dependent on the available free volume in the form of molecular sized holes in the polymer matrix and also the affinity of polymer towards water. This available free volume in turn depends on the morphology, crosslink density, and polymer structure. The water affinity of polymer depends on the hydrogen bonding sites to be present along the polymeric chains, to develop attractive forces between polymer and water molecules [39]. During water aging at 90 °C, microstructural deformation may possibly take place in the bulk of the sample, because of its exposure to high temperatures [41]. Polymer crosslinking density gets affected by this, increasing the free volume. Due to this, water molecules diffuse more into the sample aged at 90 °C compared to the sample aged at room temperature. In addition, Huner et al. have indicated that the degradation induced due to water intake in composite specimens is more predominant at high temperatures [55]. These epoxy specimens were kept for drying for a period of 24 h after 240 h of water aging. Hence, three epoxy micro-nano composite specimens: Unaged specimen, water aged specimens at room temperature (RT) and 90 °C, respectively, were finalized for further characterizations.

### 2.3. Space Charge Measurement

**Figure 3:**
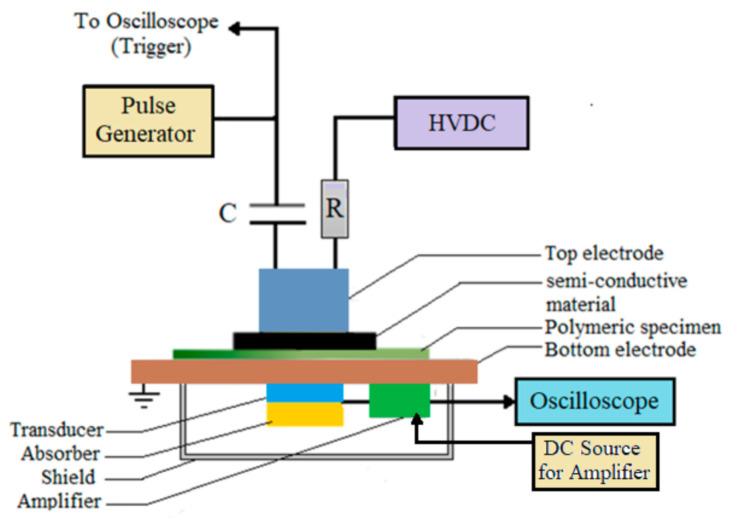
Pulsed electro acoustic (PEA) experimental setup of space charge measurement [30].

Figure 3 represents the experimental setup for the measurement of space charge adopting pulsed electro acoustic (PEA) method. In PEA method, when an electrical pulse is applied to the specimen, acoustic waves are produced at charge layers at both electrode-specimen interface and at internal charges. These acoustic signals are converted into electric signals by a piezo-electric transducer, which represents the charge distribution. This obtained electric signal after calibration gives the quantitative information about space charge density [56]. Techimp PEA System has been employed for space charge measurement. This experimental setup consists of a PEA Flat cell, a voltage pulse generator with voltage magnitude of 0–500 V, pulse width of 10 ns and frequency of 150 Hz, a high voltage variable DC source in the range of 0–30 kV, a DC source of 18–24 V to supply the amplifiers, and an oscilloscope (Tektronix, 350 MHz, 5 GS/s). The test specimen used for the study is a flat type sheet material with dimensions 40 × 40 × 1 mm^3^.

## 3. Results and Discussion

### 3.1. Impact of Gamma Irradiation on Space Charge Characteristics of Epoxy Micro-Nano Composites

**Figure 4:**
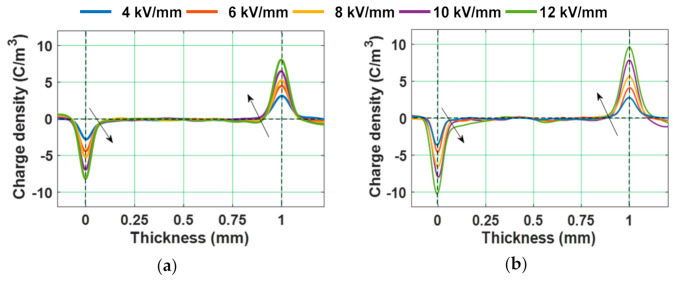
Variation in space charge density under different applied electric fields in (**a**) unaged and (**b**) 8 kGy gamma irradiated epoxy micro-nano composites [29].

Difference in the current density in the bulk volume of a dielectric material under the applied field conditions results in the formation of space charge [57]. Figure 4 represents the space charge profiles of epoxy specimens under various positive DC electric fields. The presence of homo-charge is noticed in test specimens, near the vicinity of the electrode–dielectric interface. The net space charge formation is dependent on processes such as charge injection/extraction and charge transportation. When the rate of charge injection is higher than the rate of charge transportation, it results in the formation of homo-charge. The amount of homo-charge formed near the electrode-dielectric interface increases with increase in the magnitude of applied electric field. According to the Schottky process, the charge carriers near the electrode–dielectric interface should overcome a potential barrier in order to enter into dielectric material [58]. The potential barrier decreases with the increase in the magnitude of applied electric field, making the charge injection process easier. Therefore, the amount of homo-charge formed near the interface tends to increase with increase in the applied electric field.

The magnitude of space charge accumulated at an applied electric field helps in understanding the extent of degradation of the test specimen. The mean magnitude of accumulated space charge density q(E,t) in the specimen can be calculated as shown in (3) [57].

(3) $$q\left(E,t\right)=\frac{1}{x_{1}-x_{0}}\int_{x_{0}}^{x_{1}}\left|q_{p}\left(x,t;E\right)\right|dx$$

where *x*_0_ and *x*_1_ represent the position of the electrodes (induced charges at the electrodes are neglected), and $q_{p}\left(x,t;E\right)$ is the charge density at position *x*, time *t*, and electric field applied *E*.

**Figure 5:**
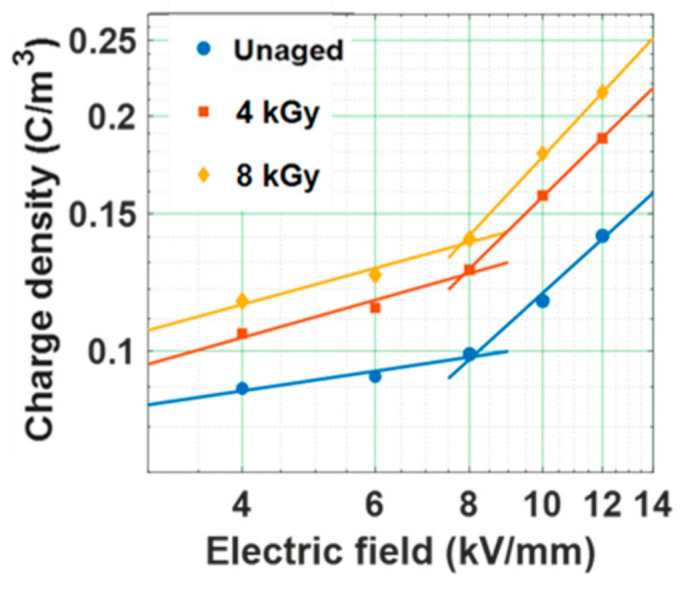
Average space charge density as a function of applied electric field in gamma irradiated samples [29].

The threshold for space charge accumulation (*E_T_*) and rate of space charge accumulation (*b*) can be calculated from the plot depicting average space charge density versus electric field applied (Figure 5). The parameters *E_T_* and *b* are not dependent on the applied electric field and the time, provided that the average space charge density is measured at the quasi-steady state condition of space charge accumulation [59]. The average charge density just after one hour of poling time at each electric field is considered for calculating *E_T_*. The slope of the fitted line above the threshold point (*E_T_*) of each sample in Figure 5, provides the parameter *b* [59]. The parameter *E_T_* is marginally higher for unaged sample rather than gamma irradiated samples (Table 1). This indicates that the field above which a significant space charge accumulation occurs tends to decrease with dosage of gamma irradiation. The parameter *b* tends to increase with increase in dosage of gamma irradiation, indicating that a relatively higher amount of space charge accumulation occurs in gamma irradiated samples than unaged samples, at electric fields higher than *E_T_*. Therefore, the decrease in *E_T_* and increase in *b* with increase in dosage of gamma irradiation reflects the possibility of early degradation of the properties of the insulating material leading to premature breakdown.

**Figure 6:**
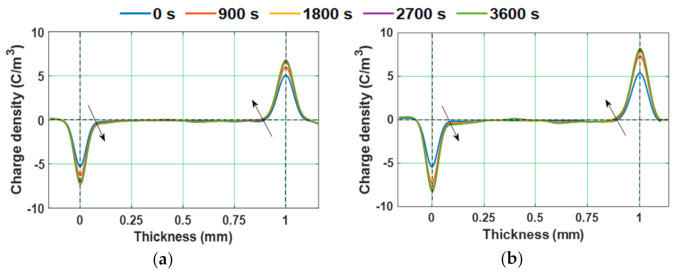
Space charge distribution during poling in (**a**) unaged and (**b**) 8 kGy gamma irradiated epoxy micro-nano composites under 10 kV positive DC voltage [29].

**Figure 7:**
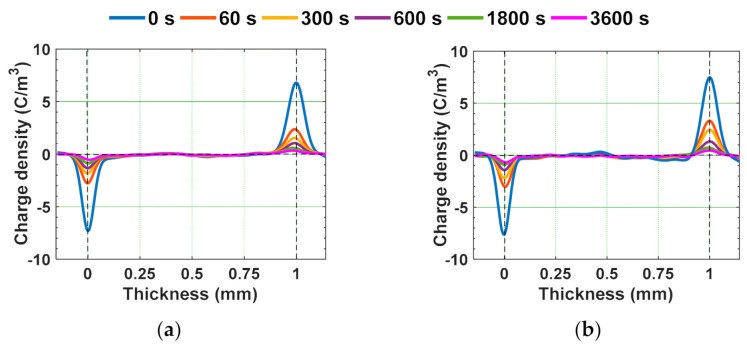
Space charge distribution during depoling of (**a**) unaged and (**b**) 8 kGy gamma irradiated epoxy micro-nano composites after reduction of 10 kV positive DC voltage [29].

Since the value of *E_T_* is noticed to be around 8 kV/mm, an electric field of 10 kV/mm is considered for further analysis. The space charge profiles of test samples at different instants of poling time, under 10 kV positive DC voltage are represented in Figure 6. An increment in the homo-charge accumulation with respect to poling time, near the electrode–dielectric interface, is observed. Figure 7 depicts the space charge characteristics of epoxy samples during depoling. The charge induced at the electrode-specimen interface is reduced instantly just after decreasing the applied voltage to zero, leaving the accumulated homo-charge.

**Figure 8:**
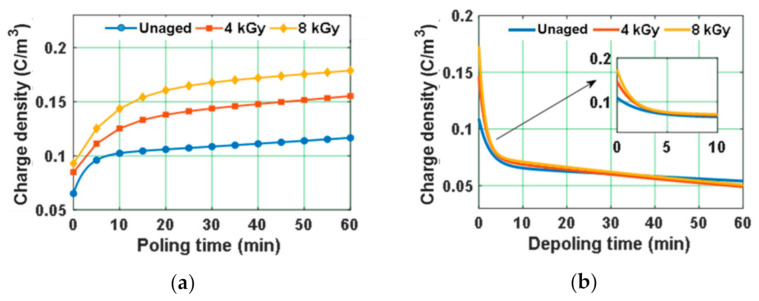
Average space charge density of unaged and gamma irradiated samples during (**a**) poling time and (**b**) depoling time [29].

The magnitude of charge density of the test samples at poling and depoling periods is depicted in Figure 8. The magnitude of space charge density is higher for gamma irradiated samples compared to unaged samples (Figure 8a). Chen et al. studied the space charge behavior of gamma irradiated polyethylene under various radiation environments and have indicated that the increase in the space charge accumulation of the gamma irradiated samples compared to unaged samples is more when irradiated in air ambience. Meanwhile, only a small amount of space charge accumulation is noticed in specimens irradiated in both nitrogen and vacuum environments [50]. Similar characteristics are noticed in the test specimens with an increase in the average space charge density with dosage of irradiation in air ambience (Figure 8a). Internal charge carriers tend to form when they gain sufficient energy to escape from valence band to conduction band. This energy to the charge carriers can be obtained by thermal, electrical, or radio-active phenomenon. Therefore, the formation of internal charge carriers or free radicals in the bulk of the gamma irradiated samples due to chain scissions and oxidation phenomena results in the increment of average space charge density.

The charge decay during depoling is faster in gamma irradiated samples than unaged ones (Figure 8b). The trapping and de-trapping phenomena are mostly related to the charge trap distribution in terms of energy depths. The charge traps in general tend to have several discrete energy depths or energy levels. However, to simplify, it is assumed that the traps are uniformly distributed across the bulk of sample and possess only two trap energy levels, i.e., shallow trap and deep trap [60]. For further simplification, the charge injection process is considered to follow the Schottky process of charge injection, and the current tends to increase exponentially with the electric field applied and tends to decay exponentially during the depoling time. Therefore, the charge decay curves during depoling were modelled as double exponential function as represented in (4).

(4) $$n\left(t\right)=n_{1}e^{-ax}+n_{2}e^{-bx}$$

where summation of *n*_1_ and *n*_2_ gives the magnitude of space charge density just before depoling, and a and b are the exponential factors which indicate the charge decay rates.

**Figure 9:**
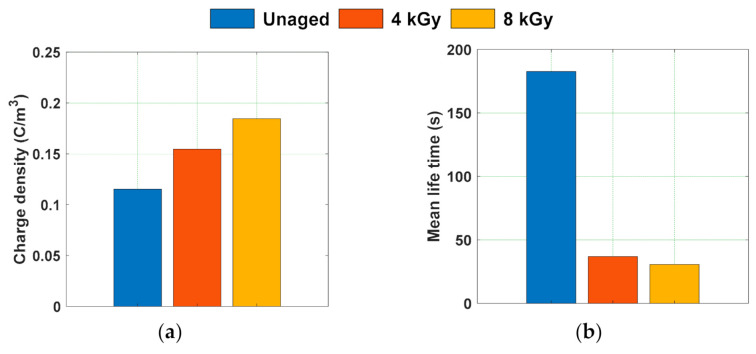
(**a**) Initial charge density just before depoling and (**b**) mean lifetime of space charge decay of gamma irradiated samples.

The parameters determined by modelling the magnitude of space charge density during depoling time are tabulated in Table 2. Zhou et al. have stated that the parameters *a*, *b*, *n*_1_, and *n*_2_ are related to microstructure of the specimen and any changes in these parameters will affect the aging process that takes place in the specimen [60]. Figure 9 depicts the magnitude of initial charge density just before depoling, and the mean lifetime of the charge decay curves that are determined from the parameters represented in Table 2. The charge decay rate “*b*” is order of 10^−4^, which reflects that the charge decay corresponding to the part *n*_2_ is almost negligible when compared to charge decay of the part *n*_1_. Hence, to compare the charge decay parameters of unaged and gamma irradiated specimens during depoling time, only the charge decay rate “*a*” is considered for calculating mean lifetime of charge decay. The mean lifetime of the charge decay is lesser in gamma irradiated epoxy samples compared to unaged sample. Lower mean life time indicates that the charge transportation rate in the bulk of the material is higher. The transportation of charge carriers depends on the depth of the charge trapping sites. Chen et al. have indicated that the carbonyl groups concentration tends to increase with radiation dose and further the carbonyl groups generate a shallow trap that can possibly assist charge transport [61]. Therefore, the faster space charge decay in the gamma irradiated samples during depoling, could be due to the formation of a relatively higher number of shallow traps, which tends to increase the rate of charge carrier transportation.

**Figure 10:**
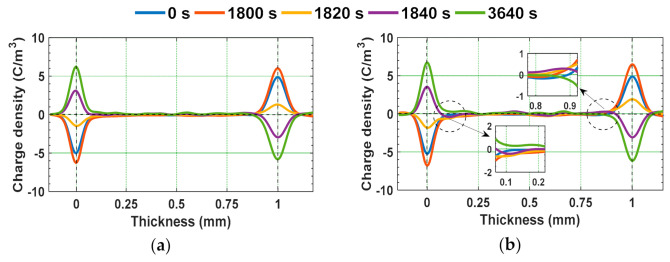
Space charge distribution under polarity reversal from positive to negative DC field stress in (**a**) virgin and (**b**) 8 kGy gamma irradiated specimens [29].

**Figure 11:**
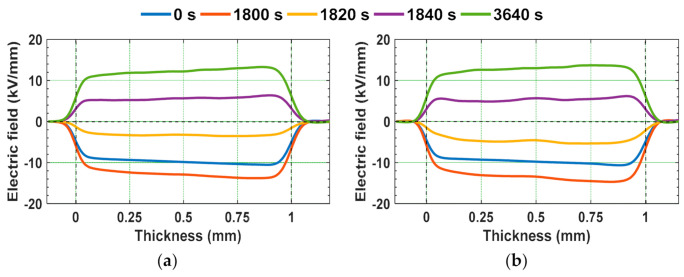
Electric field distribution under polarity reversal from positive to negative DC field stress in (**a**) virgin and (**b**) 8 kGy gamma irradiated specimens [29].

The epoxy micro-nano composites were subjected to 10 kV/mm positive DC field for 1800 s before the polarity reversal, and after polarity reversal, the samples were subjected to 10 kV/mm negative DC field for 1800 s. In between, the voltage polarity reversal time was maintained as 40 s. The space charge density and electric field distribution profiles of unaged and gamma irradiated epoxy samples during poling time and polarity reversal period are depicted in Figure 10 and Figure 11.

The accumulated homo-charge before voltage polarity reversal (at 1800 s) in Figure 10b is retained during the polarity reversal period (1800–1840 s). The field distribution is modified due to this retained space charge and this will further affect the space charge distribution after voltage polarity reversal. After the polarity reversal phenomenon, the accumulated space charge due to opposite polarity field stress first reduces the retained charge and then cancels it. Then, finally, the space charge with reversed polarity is formed at the same position by the end of poling period after reversal (at 3640 s). Wang et al. have indicated that the homo-charges injected before polarity reversal will remain in the insulating material as hetero-charges after the polarity reversal of DC field. They have indicated that the portion of charge retained after polarity reversal could possibly migrate under reversed electric field or get neutralized by the electrons injected from cathode [48]. Similar behavior is observed from Figure 10b, depicting the space charge density of 8 kGy irradiated sample during voltage polarity reversal.

Space charge presence in a dielectric can possibly lead to local electric field distortion, due to which the dielectric will experience higher electric fields than the applied electric field, in some of the regions in the bulk of the specimen. The extent of electric field distortion can be quantified by calculating the electric field enhancement factor [62]. It is represented as shown in (5).

(5) $$F=\frac{E-E_{a}}{E_{a}}\times 100$$

where $E_{a}$ represents the electric field applied (kV/mm), E is the maximum electric field in the bulk of the specimen (kV/mm), and F represents the field enhancement factor (%).

**Figure 12:**
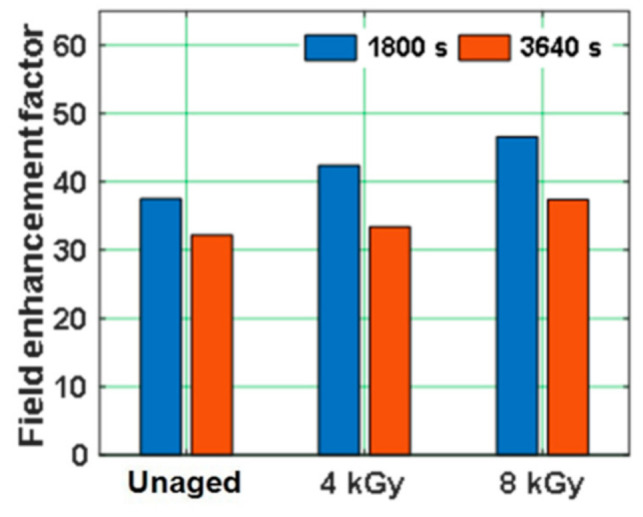
Field enhancement factor of unaged and gamma irradiated epoxy nanocomposites [29].

The electric field enhancement factor of unaged and gamma irradiated samples just before polarity reversal i.e., at 1800 s and after a poling time of 1800 s from polarity reversal i.e., at 3640 s, can be depicted in Figure 12. The electric field enhancement tends to increase with gamma radiation dose, before and after the voltage polarity reversal phenomenon.

### 3.2. Impact of Water Aging on Space Charge Characteristics of Epoxy Micro-Nanocomposites

**Figure 13:**
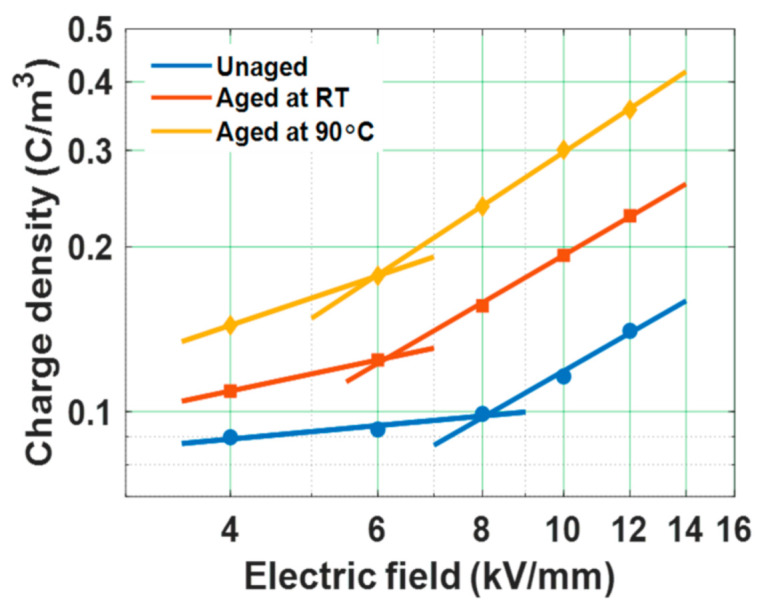
Relation between mean magnitude of space charge density and applied electric field of water aged samples [30].

The magnitude of space charge accumulation is a function of applied electric field applied across the sample. The plot depicts the dependance of the magnitude of space charge density accumulated (calculated from (3)) and applied electric field (Figure 13). The threshold electric field (*E_T_*) of the test samples tends to reduce significantly after water aging (Table 3). The rate of space charge accumulation (*b)* is noticed to increase after water aging. Decrement in the value of *E_T_* with water ageing, confirms the decrease in the potential barrier (as per the Schottky process of charge injection [58]), resulting in easier charge injection process. Hence, the reduction in *E_T_* and increment in parameter *b* after water aging leads to the early deterioration of the epoxy specimens.

**Figure 14:**
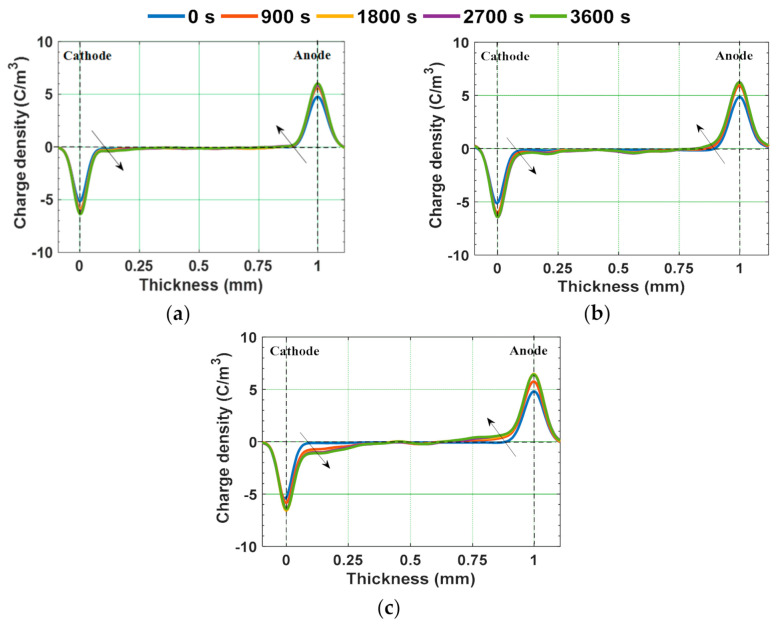
Space charge profiles during poling in (**a**) unaged, (**b**) water aged sample at RT, and (**c**) water aged sample at 90 °C [30].

**Figure 15:**
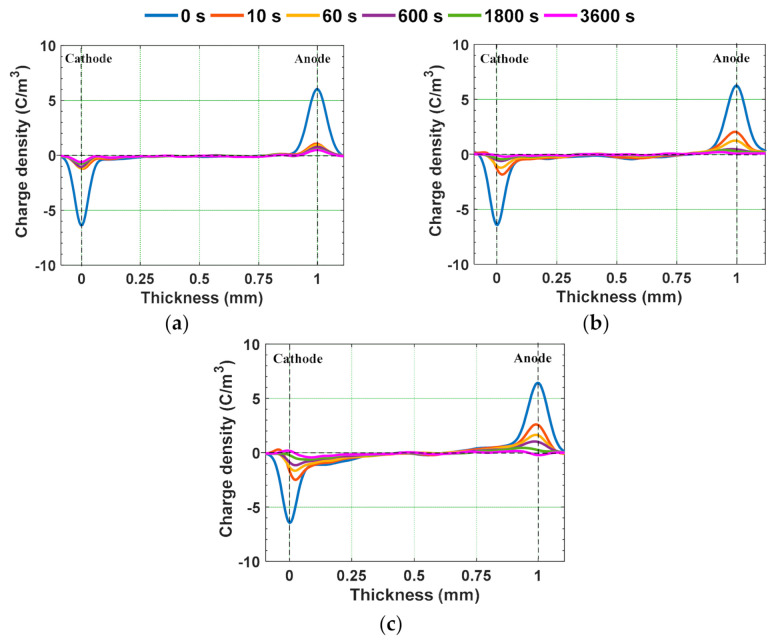
Space charge profiles during depoling in (**a**) unaged, (**b**) water aged sample at RT, and (**c**) water aged sample at 90 °C [30].

**Figure 16:**
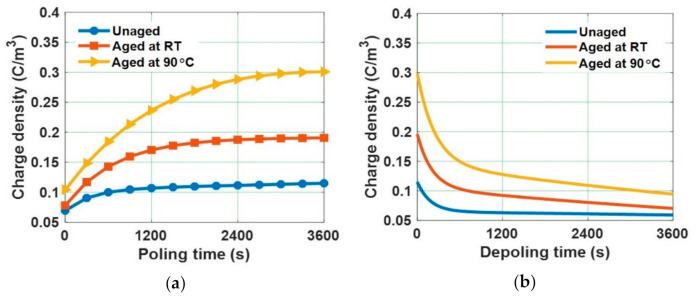
Mean value of absolute space charge density in water aged samples during (**a**) poling time and (**b**) depoling time [30].

Since, the value of *E_T_* of the water aged samples is lesser than 8 kV/mm, 10 kV/mm is considered as applied electric field. Homo-charge formation is observed in water samples, indicating that the charge injection rate is more than the charge transportation rate [58]. Homo-charge accumulation is increased more significantly in water aged samples compared to unaged samples (Figure 14). Figure 15 depicts the space charge characteristics of water aged epoxy samples at different instants of depoling time. The charges induced at the electrode-sample interface has reduced as soon as applied voltage is decreased to zero, leaving only the space charge. The magnitude of space charge density in the water aged samples during both the poling and depoling periods are represented in Figure 16. The magnitude of charge density during poling period is higher in water aged samples compared to unaged sample (Figure 16a). Fabiani et al. have stated that the increment in the amount of water absorption into the volume of the sample results in the increment of space charge accumulation [63]. The potential barrier of charge injection at the electrode-specimen interface tends to decrease with the diffusion of water molecules, allowing more amount of charge to inject into the volume of the specimen [63]. Therefore, the mean magnitude of accumulated charge density is directly in proportion to the amount of water diffusion into the specimen.

**Figure 17:**
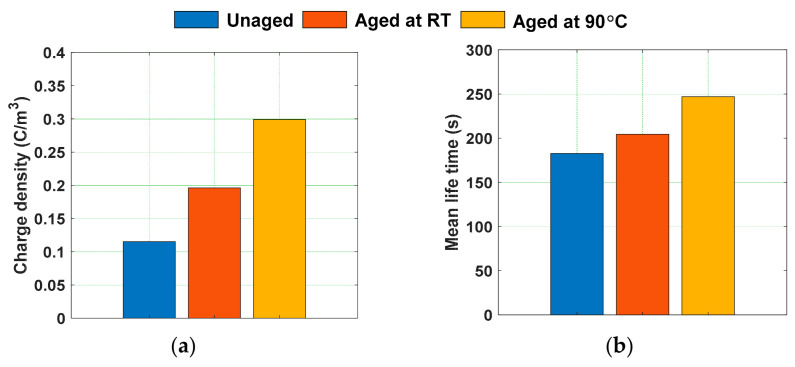
(**a**) Initial charge density just before depoling and (**b**) mean lifetime of space charge decay of water aged samples [30].

The space charge decay curves during depoling period were modelled with a double exponential function as represented in (4). The parameters obtained by modelling the magnitude of space charge density have been tabulated in Table 4. Figure 17 shows the magnitude of initial charge density just before depoling period and the mean lifetime of the space charge decay curves calculated from the parameters represented in Table 4. The mean lifetime of the space charge decay is more in 90 °C water aged samples, followed by the room temperature water aged sample and the unaged sample, respectively. The charge carriers that enter into the bulk of the sample through charge injection are trapped by the localized states, which originate due to crystal defects, matrix–filler interface, and catalyst residues [64]. The microstructural deformation in the water aged samples at high temperatures will lead to the increment of localized trapping sites resulting in the reduction of the space charge decay rate.

**Figure 18:**
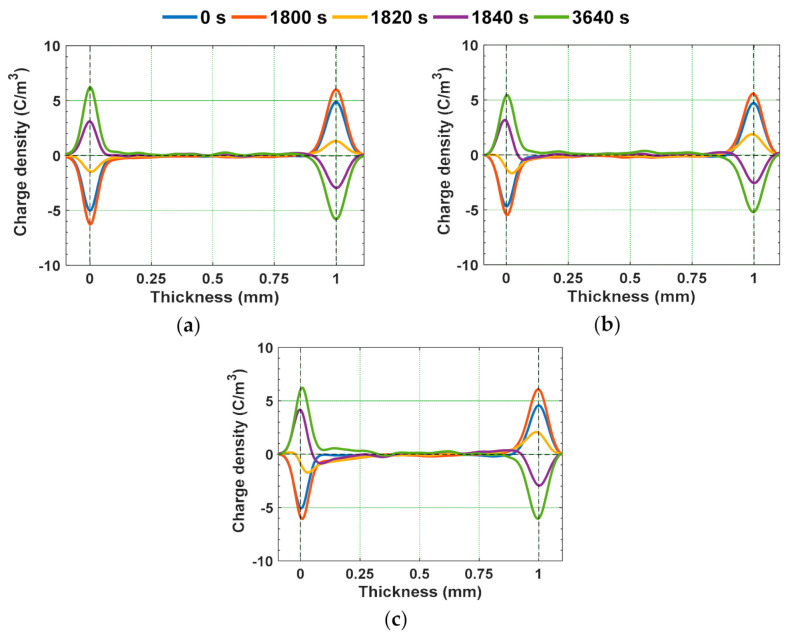
Space charge characteristics under voltage polarity reversal in (**a**) unaged specimen, (**b**) water aged specimen at RT, and (**c**) water aged specimen at 90 °C [30].

**Figure 19:**
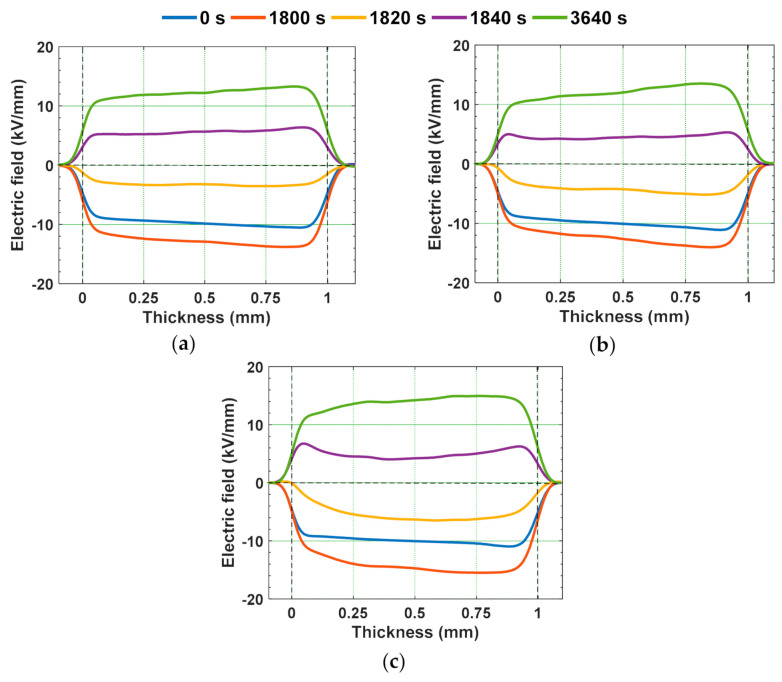
Electric field characteristics under voltage polarity reversal in (**a**) unaged specimen, (**b**) water aged specimen at RT, and (**c**) water aged specimen at 90 °C [30].

**Figure 20:**
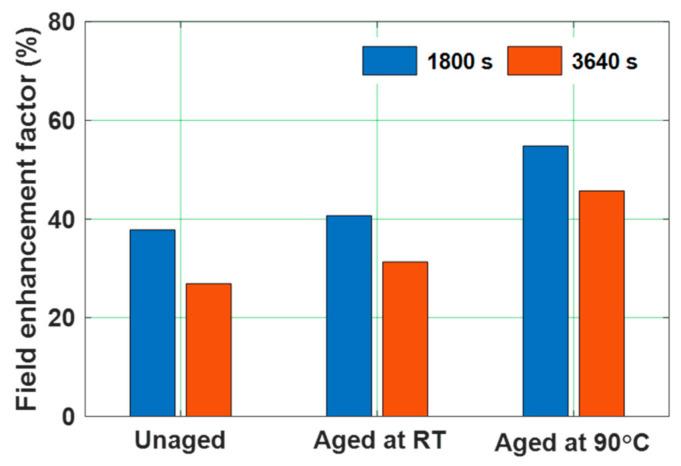
Field enhancement factor of unaged and water aged specimens [30].

The water aged epoxy specimens were subjected to voltage polarity reversal, by maintaining the polarity reversal time and voltage same as that of the polarity reversal phenomenon performed for gamma irradiated specimens mentioned above. Figure 18 and Figure 19 represent the space charge density and electric field distribution of water aged epoxy samples. Similar to that of gamma irradiated specimens under polarity reversal, some portion of accumulated homo charge in water aged before polarity reversal is remained after the change in the voltage polarity also. It is because the polarity reversal time is lesser than the time required for the accumulated space charge to decay. The remained charge after polarity reversal acts as hetero-charge and tends to alter the local electric field distribution [48]. This can either increase or decrease the electric field, depending on the type of the space charge accumulated. Homo-charge accumulation near the electrode-specimen interface reduces the local electric field, whereas the hetero-charges enhance the local electric field. The retained homo-charge in the water aged epoxy specimens, just after polarity reversal i.e., 1840 s, acts as hetero-charge, enhancing the local electric field near the electrode-sample interface. This electric field distortion can be quantified by calculating the electric field enhancement factor as shown in (5). The electric field enhancement factor of water aged specimens at the instant of 1800 s and 3640 s is represented in Figure 20. The electric field enhancement factor is higher in the 90 °C water aged sample compared to the water aged sample at room temperature. The water aged samples are subjected to higher field distortion than unaged samples. This higher field distortion in 90 °C water aged sample can possibly lead to the reduction in the insulator reliability and life span compared to unaged one.

### 3.3. Comparitive Study on the Impact of Gamma Irradiation and Water Aging of Space Charge Characteristics of Epoxy Micro-Nanocomposites

Homo-charge formation due to charge injection phenomenon has been noticed in the space charge profile of the unaged epoxy micro-nano composite specimen. This homo-charge formation is marginally increased in case of gamma irradiated samples than unaged sample. Whereas a significant increment in the formation of homo-charge near sample-electrode interface is noticed in water aged samples compared to the unaged sample. Moreover, at high temperature, where the water diffusion coefficient is higher than the diffusion coefficient at room temperature, the charge accumulation is observed to be higher. The mean magnitude of accumulated space charge density also reflected the same trend. In polymeric composite samples, incomplete dangling chain ends that originate near matrix–filler interfacial region due to catalyst residues, crystal defects, and aging mechanisms results in increment of localized trapping sites [64]. With aging mechanism, due to the deterioration of the molecular structure of the sample, the number of localized trapping sites increases, eventually resulting in higher space charge accumulation. The amount of charge density accumulated tends to increase with increase in the magnitude of DC applied electric field in all the test conditions. The threshold electric field (*E_T_*) tends to decrease and rate of space charge accumulation (*b*) increases with increase in gamma irradiation dose as well as increase in diffusion coefficient of water. Significant reduction parameter *E_T_* is observed in water aged samples and only a marginal reduction in *E_T_* is observed with gamma irradiated samples.

One significant observation noticed form the comparison of two different aging procedures is that the decay rate of the accumulated space charge is higher in gamma irradiated epoxy samples compared to the unaged sample, whereas the space charge decay rate has reduced after water aging. The transportation of charge carriers is influenced by the depth of the charge trapping sites. The formation of carbonyl groups due to radiation-induced chemical reactions generate a tend to increase shallow trap density assisting charge transport, further leading to increased charge decay rate. The microstructural deformation in the water aged samples at high temperatures will lead to the increase of localized trapping sites resulting in the lowering the space charge decay rate.

Space charge profile of all the test samples during voltage polarity reversal reflected a portion of homo-charge accumulated before polarity reversal remained as hetero-charge just after polarity reversal. This remaining charge led to the distortion of local electric field. The electric field enhancement factor of aged samples tends to increase with the increase in radiation dose as well as diffusion coefficient, indicating aged samples are subjected to higher field distortion. This higher field distortions in aged epoxy samples minimizes the reliability and life span of the insulating material [65]. Hence, the field enhancement during the polarity reversal phenomenon is influenced by the pre-existing space charge (space charge accumulated just before polarity reversal) [66].

## 4. Conclusions and Future Scope

Epoxy nanocomposites are the most promising family of materials gaining much attention for the past few decades, because of their unique feature in achieving the desired properties in wide range of applications. Recent studies have proved that the addition of micro-silica particles to base epoxy matrix have successfully enhanced the mechanical properties of epoxy micro composites. Reinforcement of small wt.% of nanofillers to these micro composites have enhanced electrical as well as thermal properties. Hence, the epoxy-micro-nano composites can possess the desired properties of both micro and nanocomposites.

Ion-trapping nano particles, which possess good oxidation resistance and high ion trapping ability, are being chosen as fillers along with silica micro fillers in epoxy micro-nano composite material for improving the reliability of electrical insulation structures. These epoxy micro-nano composites were subjected to different aging procedures (gamma irradiation and water aging) and their space charge behavior with respect to aging is analyzed. The important conclusions were arrived are as follows:

- Homo-charge formation due to charge injection phenomenon has been noticed in the space charge profile of both the unaged and aged epoxy micro-nano composite specimen.
- The threshold electric field (*E_T_*) tends to decrease and rate of space charge accumulation (*b*) increases with increase in gamma irradiation dose as well as increment in diffusion coefficient of water
- Magnitude of accumulated space charge density is marginally increased in case of gamma irradiated samples than unaged sample, whereas a significant increment is noticed in water aged samples compared to unaged sample.
- The decay rate of the accumulated space charge is higher in gamma irradiated epoxy samples compared to unaged sample, whereas the space charge decay rate has reduced after water aging.
- Space charge profile of all the test samples during voltage polarity reversal reflected a portion of homo-charge accumulated before polarity reversal remained as hetero-charge just after polarity reversal.
- The electric field enhancement factor of aged samples tends to increase with increase in radiation dose as well as diffusion coefficient, indicating aged samples are subjected to higher field distortion

Finally, it can be concluded that the incorporation of ion-trapping nano particles in epoxy micro-nano composites for power apparatus application is truly promising. However, more research work needs to be carried out in future by diversifying the nanoparticles used and by subjecting these epoxy micro-nano composites to different aging conditions, in order to design a suitable material that can overcome major challenges due to aging conditions in industry and to be potentially applicable in future electrical insulation structures.

## Figures and Tables

**Table 1:** Parameter values of unaged and gamma irradiated samples [29].

Specimen	*E_T_* (kV/mm^−1^)	*b* (µCV^−1^ m^−2^)
Unaged	8.08	0.876
4 kGy	7.88	0.952
8 kGy	7.83	1.038

**Table 2:** Modelling parameters of mean magnitude of charge density during depoling time.

Specimen	*n*_1_	*a*	*n*_2_	*b*
Unaged	0.0502	0.00548	0.0649	2.26 × 10^−5^
4 kGy	0.1190	0.02708	0.0356	2.37 × 10^−4^
8 kGy	0.1288	0.03261	0.0557	2.25 × 10^−4^

**Table 3:** Space charge parameter values of unaged and water aged samples [30].

Specimen	*E_T_* (kV/mm^−1^)	*b* (µCV^−1^ m^−2^)
Unaged	8.03	0.875
Aged at RT	6.26	0.891
Aged at 90 °C	5.94	1.008

**Table 4:** Modelling parameters of magnitude of charge density during depoling time [30].

Specimen	*n*_1_	*a*	*n*_2_	*b*
Unaged	0.0502	0.00548	0.0649	2.26 × 10^−5^
Aged at RT	0.0908	0.00489	0.1053	11.23 × 10^−5^
Aged at 90 °C	0.1524	0.00405	0.1465	12.21 × 10^−5^

## Data Availability

The data that support the findings of this study are available from the corresponding author upon reasonable request.
